# Large Language Models in Medical Image Analysis: A Systematic Survey and Future Directions

**DOI:** 10.3390/bioengineering12080818

**Published:** 2025-07-29

**Authors:** Bushra Urooj, Muhammad Fayaz, Shafqat Ali, L. Minh Dang, Kyung Won Kim

**Affiliations:** 1 Department of Medical Science, Asan Medical Institute of Convergence Science and Technology, University of Ulsan College of Medicine, Seoul 05505, Republic of Korea; urooj@mail.ulsan.ac.kr; 2 Department of Computer Science and Engineering, Sejong University, Seoul 05006, Republic of Korea; muhammadfayaz@sju.ac.kr; 3 Department of Software Engineering, Sejong University, Seoul 05006, Republic of Korea; shafqatali@sju.ac.kr; 4 The Institute of Research and Development, Duy Tan University, Da Nang 550000, Vietnam; 5 Faculty of Information Technology, Duy Tan University, Da Nang 550000, Vietnam

**Keywords:** large language models, medical image analysis, x-stage tuning, disease classification, medical report generation, visual question answering, multimodal learning

## Abstract

The integration of vision and language processing into a cohesive system has already shown promise with the application of large language models (LLMs) in medical image analysis. Their capabilities encompass the generation of medical reports, disease classification, visual question answering, and segmentation, providing yet another approach to interpreting multimodal data. This survey aims to compile all known applications of LLMs in the medical image analysis field, spotlighting their promises alongside critical challenges and future avenues. We introduce the concept of X-stage tuning which serves as a framework for LLMs fine-tuning across multiple stages: zero stage, one stage, and multi-stage, wherein each stage corresponds to task complexity and available data. The survey describes issues like sparsity of data, hallucination in outputs, privacy issues, and the requirement for dynamic knowledge updating. Alongside these, we cover prospective features including integration of LLMs with decision support systems, multimodal learning, and federated learning for privacy-preserving model training. The goal of this work is to provide structured guidance to the targeted audience, demystifying the prospects of LLMs in medical image analysis.

## 1. Introduction

Medical imaging serves as a fundamental pillar in modern healthcare, delivering critical insights into disease detection, treatment planning, and patient monitoring. In clinical practice, medical tasks such as image classification, segmentation, automated report generation, and registration have evolved into crucial elements [1]. While traditional machine learning techniques established the foundation for automation in these domains, they face significant challenges in processing the diverse and complex nature of clinical imaging data [2,3]. Additionally, the remarkable progress of modern artificial intelligence techniques has resulted in substantial improvements across various medical image analysis tasks [4]. However, these models typically require specialized training and large annotated datasets, restricting their scalability and generalization across different clinical applications [5,6,7,8].

The emergence of cutting-edge large language models has revolutionized multiple domains, particularly in natural language processing (NLP), owing to their powerful generalization abilities and multimodal input processing capabilities [9]. In medical imaging, LLMs, especially vision-language models, have shown considerable potential to consolidate diverse tasks within a unified framework. In the context of this review, “productivity” refers to the ability of large language models (LLMs) to efficiently and effectively perform medical image analysis tasks, such as image classification, medical report generation, and visual question answering. It is a measure of how well these models can process and integrate medical image data with textual descriptions, providing valuable outputs that assist healthcare professionals in decision-making. Productivity is assessed in terms of both the speed at which these tasks are completed and the accuracy and reliability of the model’s outputs.

**Figure 1:**
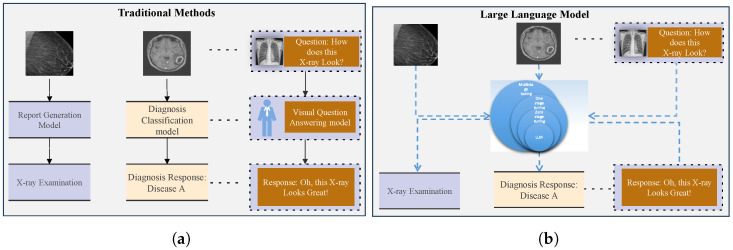
Traditional approaches require task-specific models to solve different tasks, as shown in (**a**). In contrast, LLMs can employ a unified model to handle multiple tasks, as shown in (**b**).

With proper prompting, LLMs can analyze visual content, produce diagnostic insights, and execute various downstream tasks, potentially alleviating the workload of healthcare professionals while enhancing clinical efficiency. Traditional machine learning and LLM algorithms are generally presented in Figure 1, in which (a) represents traditional methods and (b) illustrates medical image analysis through a large language model.

Despite increasing enthusiasm for applying LLMs to medical image analysis, a thorough and well-organized synthesis of this field is still missing. This gap hinders researchers’ ability to evaluate current progress, recognize challenges, and discover opportunities in this quickly advancing field. To bridge this gap, we conducted a systematic review documenting the current status of LLMs in medical image analysis. To offer a comprehensive perspective, we introduced an x-stage tuning paradigm encompassing zero-stage, one-stage, and multi-stage tuning, presenting a unified framework for investigating LLM applications in this domain. Several recent surveys have examined the use of large language models in healthcare, providing valuable insights into applications such as multimodal medical question answering, clinical decision support, and Transformer architectures [10]. However, these surveys are often limited in scope focusing either on specific tasks or predominantly on text-based applications and do not extend their analysis to vision-language models or practical adaptation strategies in medical image analysis. Additionally, none provide a systematic, stage-based tuning framework to help practitioners understand how foundation models can be effectively adapted for different imaging tasks [11,12]. Our work addresses these gaps by offering a structured, task-oriented perspective on model tuning for medical image analysis.

The proposed X-stage tuning paradigm introduces a novel conceptual framework that categorizes adaptation strategies based on tuning complexity and domain alignment. This structure enables direct comparison of tuning approaches across various medical imaging tasks and datasets, supporting researchers and developers in selecting suitable strategies for model deployment under different constraints.

- Zero-stage tuning refers to applying LLMs to medical image tasks without any additional tuning, enabling direct use with minimal resources and data.
- One-stage tuning involves fine-tuning LLMs using domain-specific medical image data to capture relevant features and improve performance.
- Multi-stage tuning includes a sequence of tuning phases, often leveraging both domain-agnostic and domain-specific datasets to build more robust and adaptable models [13,14].

Furthermore, we identify current limitations and future directions in this domain, offering critical insights and practical guidance to researchers and developers. This survey is especially timely as the field transitions from developing general-purpose foundation models to implementing domain-specific solutions in regulated clinical environments. The insights provided here are intended to support AI researchers working on model design, healthcare developers integrating AI into clinical tools, and clinicians seeking to understand the practical implications of using large language models in medical imaging workflows.

The contributions of this work are summarized as follows:

- We present a detailed synthesis of the current applications of LLMs across medical imaging tasks, including automated radiology report generation, disease classification, visual question answering (VQA), and visual grounding.
- We propose an x-stage tuning paradigm to systematically categorize current approaches and highlight their differences, use cases, and limitations.
- We perform a thorough analysis of zero-stage, one-stage, and multi-stage fine-tuning strategies, providing insights into their comparative performance on various tasks and datasets.
- We explore current trends, unresolved challenges, and promising avenues for future research in this rapidly evolving field.

This paper follows an orderly structure: Section 2 provides background on LLMs, the x-stage tuning paradigm, and medical image tasks. Section 3 explores the x-stage tuning framework in detail, while experimental results are discussed in Section 4. Section 5 discusses challenges in applying LLMs to medical image analysis. Section 6 outlines future research directions and Section 7 concludes the paper with key findings and next steps.

## 2. Background

This section outlines essential background information regarding the concept of staged fine-tuning, along with the key medical image analysis tasks handled by LLMs. To properly utilize LLMs in medical image analysis, one must first grasp how staged fine-tuning works and how LLMs can be customized for specific tasks.

### 2.1. Staged Fine-Tuning

Within clinical imaging applications, progressive model adaptation is a fundamental technique for customizing LLMs to specialized tasks. By gradually adjusting model parameters through multiple stages, this approach allows for better performance when addressing specialized diagnostic requirements with available imaging data.

**Figure 2:**
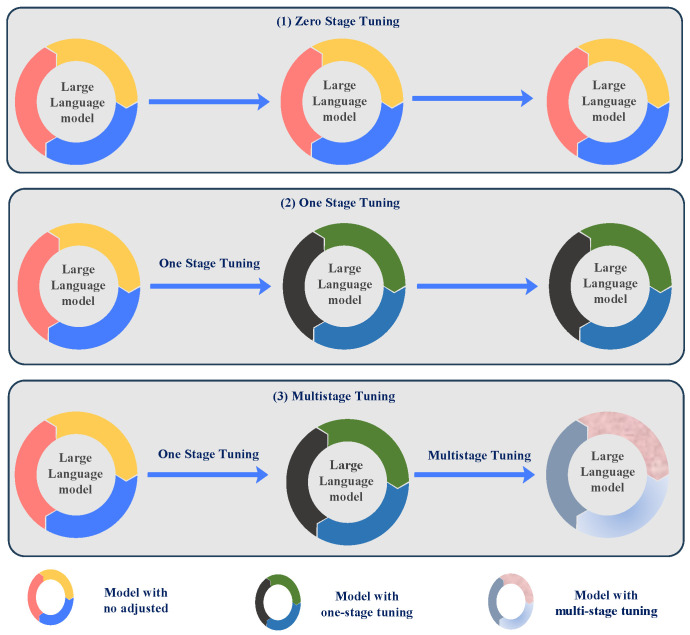
Diagram of the model tuning stage in x-stage tuning. Zero-stage tuning represents no adjustment of the model, one-stage tuning is tuning the model for one stage, and multi-stage tuning is tuning the model for more than one stage.

- Zero-stage fine-tuning: This method applies LLMs directly to medical image tasks without fine-tuning the model using specialized medical datasets, as illustrated in Figure 2(1). The model utilizes its pre-trained knowledge from a broad corpus to perform the task, enabling quick deployment but potentially compromising optimal performance for medical-specific tasks. The zero-stage fine-tuning process is expressed as
  $$\hat{Y}=M_{\mathrm{freeze}}\left(X_{\mathrm{img}},X_{\mathrm{prompt}}\right)$$
  where $\hat{Y}$ is the output generated by the model, $X_{\mathrm{img}}$ is the medical image input, and $X_{\mathrm{prompt}}$ is the associated text prompt. The notation $M_{\mathrm{freeze}}$ indicates that the model’s parameters are not adjusted during this stage.
- One-stage fine-tuning: In this approach, LLMs are fine-tuned using domain-specific medical image data to modify the model for medical imaging features, as presented in Figure 2(2). This stage results in improved performance for specific tasks, such as medical image classification or report generation [15]. The one-stage fine-tuning process is given by
  $$\hat{Y}=M_{\mathrm{tune}}\left(X_{\mathrm{img}},X_{\mathrm{prompt}}\right)$$
  where $M_{\mathrm{tune}}$ indicates that the model is fine-tuned on medical image data to optimize its performance for specific tasks.
- Multi-stage fine-tuning: The model’s refinement through multiple stages advances progressively by combining domain-specific data with general-purpose data at each stage of enhancement. This approach enhances the robustness and versatility of the model, particularly for complex medical image analysis tasks, as shown in Figure 2(3). The multi-stage fine-tuning process is represented as
  $$M_{{\mathrm{stage}}_{1}}\;\mathrm{tuning}\;⟶M_{{\mathrm{stage}}_{2}}\;\mathrm{tuning}\;⟶\cdots ⟶M_{{\mathrm{stage}}_{n}}\;\mathrm{tuning}$$
  The final model after all stages is expressed as
  $$\hat{Y}=M_{\mathrm{final}}\left(X_{\mathrm{img}},X_{\mathrm{prompt}}\right)$$
  Through multi-stage fine-tuning, the model handles diverse data across different stages, which makes it highly effective for complex multi-dimensional medical image tasks [14].

### 2.2. Medical Image Analysis Tasks

**Figure 3:**
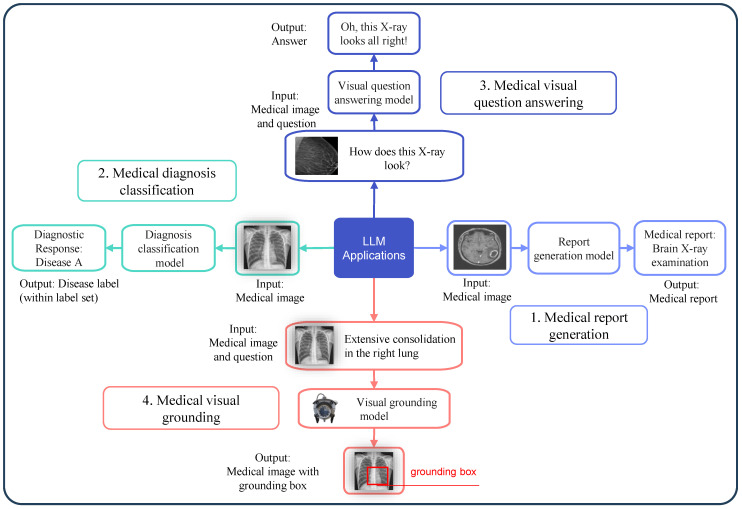
Medical image tasks with the application of an LLM.

LLMs have shown great promise in solving several key tasks within medical image analysis. These applications involve interpreting medical images and extracting valuable information as presented in Figure 3 that supports clinical decision-making [16,17]. Below are the descriptions of four common medical image analysis tasks that LLMs can help address: report generation from images, disease classification, answering questions based on images, and identifying specific regions in images based on text descriptions.

#### 2.2.1. Report Generation from Images

This task involves the automated creation of clinical reports from diagnostic imaging data, as shown in Figure 3(1), encompassing X-rays, MRIs, and CT scans. The model generates a detailed textual report describing any conditions present from input images [18,19,20]. This can be mathematically represented as

$$R=M(X_{\mathrm{img}})$$

where *R* is the generated medical report and $X_{\mathrm{img}}$ is the input medical image. Report generation evaluation typically utilizes BLEU [21] and ROUGE [21,22], as well as Perplexity and F1-score as quality metrics for the generated reports.

#### 2.2.2. Disease Classification

This task involves classifying medical images into different categories by identifying the presence of disease as presented in Figure 3(2). The model outputs a classification label, which can be represented as

$$L=M(X_{\mathrm{img}})$$

where *L* denotes the label from a set of possible diagnostic categories and $X_{\mathrm{img}}$ is the medical image. This task utilizes accuracy, together with the F1 score, along with the Area Under the Curve (AUC) as key performance metrics.

#### 2.2.3. Image-Based Question Answering (VQA)

In this task, a model answers questions related to medical images, as depicted in Figure 3(3). Given an image and a question, such as “What condition is shown in the MRI?”, the model provides an answer based on the visual content [23,24,25]. The relationship is expressed as

$$A=M(X_{\mathrm{img}},Q)$$

where *A* is the model’s answer, $X_{\mathrm{img}}$ is the medical image, and *Q* is the question. Metrics like accuracy (ACC), mean average precision (mAP), and BLEU are used to assess performance on this task.

#### 2.2.4. Image-Text Grounding

The task of grounding requires systems to both identify locations in medical images and establish their precise positions according to text descriptions, as illustrated in Figure 3(4). For example, a model might be tasked with identifying and highlighting a tumor in an X-ray based on a prompt. This task is mathematically represented as

$$G=M(X_{\mathrm{img}},P)$$

where *G* is the generated grounding box, $X_{\mathrm{img}}$ is the medical image, and *P* is the phrase describing the region of interest. Various metrics, such as Intersection over Union (IoU), grounding error, and Dice coefficient, measure the performance of grounding tasks.

### 2.3. What Did the Previous Review on Medical Images Discuss?

With the significant progress in machine learning, particularly deep learning, its uses in medical image processing have evolved considerably. Litjens et al. [3] offered an early synthesis of how deep learning methods influenced medical image analysis, emphasising the substantial contribution of convolutional neural networks (CNNs) and related architectures in advancing the field. Their work also outlined specific clinical applications where deep learning models have shown promise, including disease classification, organ segmentation [26,27], and tumor detection.

Following this foundational work, recent surveys, such as the one by Shamshad et al. [28], have concentrated on the evolution of Transformers in medical image processing. Their work categorized applications of Transformers across different medical domains, identified the key challenges they faced, and proposed insights on addressing these obstacles. In addition, other studies have reviewed deep learning applications across various clinical domains, such as the analysis of chest X-rays, retinal fundus images, and cardiac CT scans [29]. These efforts have served as a pivotal factor in the growth of AI technologies in the healthcare [30] sector and facilitated the initial adoption of deep learning models in clinical settings.

While these reviews have contributed significantly to the field, there remain significant obstacles, including limited datasets and privacy issues around medical data [31]. Chlap et al. [32] summarized key data augmentation techniques used to address these issues in diagnostic imaging systems, including computed tomography and magnetic resonance imaging. Morid et al. [33] focused on the application of knowledge transfer approaches in medical image analysis, outlining various strategies and their applicability in specific clinical tasks. Xie et al. [34] reviewed the infusion of domain knowledge into machine learning models and categorised research based on clinical areas and integration techniques. Moreover, researchers have introduced three approaches, namely knowledge transfer, multiple-instance learning, and learning with partial supervision, to address data scarcity issues [35,36,37].

In addition to data-related challenges, the processing of 3D medical images and improving model interpretability are also critical in clinical settings. Singh et al. [38] conducted a systematic review of 3D deep learning models for medical image applications. These models are crucial for extracting features from complex three-dimensional medical data and ensuring safer clinical applications. Furthermore, studies have explored interpretable AI methods in medical image processing, categorizing approaches, and evaluating their potential to make AI more transparent and trustworthy for healthcare professionals [39,40].

Previous surveys have addressed deep learning [3] and transformer-based methods [28] in medical imaging, focusing mainly on CNN architectures, vision-only transformers, or text-based clinical NLP models. However, these studies lack comprehensive treatment of vision-language large models (VLMs) and do not introduce structured tuning paradigms. Furthermore, existing reviews often neglect task-specific evaluation synthesis, especially across VQA, report generation, classification, and grounding. Our work fills these gaps by providing a novel tuning framework and a unified view of task-wise performance and metrics.

### 2.4. Why a Survey on LLM for Medical Images?

While deep learning methods, particularly CNNs, have been extensively studied and applied to medical image analysis, this survey seeks to address the emerging literature gap concerning how LLMs contribute to analyzing medical images. As deep learning continues to advance, the increasing computational power and volume of data have enabled the creation of increasingly sophisticated network architectures. These models integrate a vast amount of contextual knowledge and uncover deeper feature representations, which have notably improved diagnostic accuracy and clinical decision-making [41,42].

The versatility of LLMs, driven by their capacity for cross-task generalization and multi-task learning, sets them apart from smaller, task-specific models. Their ability to process various medical imaging problems, including automated lesion detection, tissue segmentation [43], and diagnosis classification [44], makes them powerful tools in clinical applications. Specifically, trained on large datasets, LLMs can identify semantic relations and underlying biomedical patterns, which is an essential asset in medical imaging scenarios because annotated data are scarce.

Nevertheless, a range of challenges that should be addressed remains despite the impressive achievements of LLMs in the sphere of medical image analysis. These are the limits in datasets and metrics, issues of knowledge updating, and the necessity of multilingual models. Moreover, it has some uncertainties about how Chain-of-Thought (CoT) reasoning would be applied, the potential dangers of hallucination in the model outputs, and the safeguarding of confidential medical information.

This review is intended to give an in-depth discussion of the current LLM method in medical image analysis in light of its existing constraints. The article describes the obstacles to LLMs in this field in detail and proposes directions of future studies. In solving these problems, we would hope to offer helpful advice to researchers who deal with medical imagery and to discover other research directions that can stretch the limitations of the area further, leading to more development in medical image analytics with LLMs.

## 3. X-Stage Tuning Paradigm

The X-Stage Tuning Paradigm is an innovative framework for optimizing large language models (LLMs) used in medical image analysis. This framework categorizes the fine-tuning process into distinct stages: zero-stage fine-tuning, one-stage fine-tuning, and multi-stage fine-tuning. Each stage offers a distinct strategy for tailoring pre-trained models to specialized tasks, as well as specific adaptation methods that depend on the data characteristics and performance targets. This section examines each of these stages in detail, highlighting their respective strengths and challenges. Table 1 summarizes various applications of LLMs in medical image analysis, detailing key tasks and associated challenges in each domain. It provides an overview of how LLMs are being utilized across different medical imaging tasks, as well as the primary obstacles encountered in each application [45,46,47].

We introduce a novel conceptual framework, called X-stage tuning, to categorize the adaptation of LLMs in medical image analysis. This framework consists of:

- Zero-stage tuning: No fine-tuning; direct model use;
- One-stage tuning: Task-specific tuning using medical data;
- Multi-stage tuning: Sequential adaptation combining general and domain-specific knowledge.

This is the first survey to organize adaptation strategies in this staged format, providing a practical guide for researchers selecting tuning strategies based on data availability, model complexity, and task specificity. The X-stage paradigm not only reorganizes known methods but also enables structured comparison and deployment guidance, which is missing in prior literature.

### 3.1. Zero-Stage Fine-Tuning

**Figure 4:**
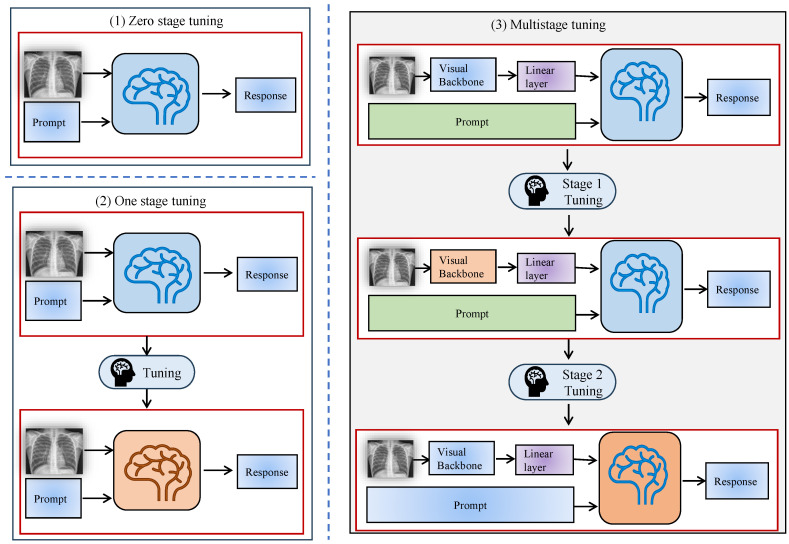
X-stage tuning diagram. (**1**) Zero-stage tuning. (**2**) One-stage tuning. (**3**) Multi-stage tuning.

Zero-stage fine-tuning refers to applying a pre-trained LLM directly to medical image tasks without modifying the model’s parameters. This stage leverages the model’s pre-existing knowledge from large, general-purpose datasets, such as image-text pairs or other natural language corpora, to perform medical image analysis tasks. Zero-stage tuning is shown in Figure 4(1). Zero-stage fine-tuning is advantageous for rapid deployment and minimizing data requirements, as it does not require labeled medical datasets or any additional training for specific tasks [48].

While zero-stage fine-tuning is efficient in terms of time and resources, it has limitations in achieving optimal performance for complex medical tasks. This is because the model has not been tailored to recognize domain-specific features and nuances in medical images, which can lead to suboptimal results when handling tasks that demand specialized medical knowledge [49,50].

### 3.2. One-Stage Fine-Tuning

The one-stage adaptation process focuses on optimizing the parameters of the pre-trained model with domain-specific medical image data. At this stage, the model undergoes task-specific training on datasets, enabling it to learn features relevant to the medical domain and enhance its performance on targeted tasks, such as disease classification, medical report generation, or image segmentation. One-stage fine-tuning is particularly useful for adapting general-purpose models to medical applications by introducing task-specific data into the training process.

This stage generally demands a substantial amount of annotated data for model training. One-stage fine-tuning needs high-quality annotated datasets to achieve successful outcomes. The model gains an enhanced understanding of medical image complexities through one-stage fine-tuning, as shown in Figure 4(2), which leads to better accuracy and result specificity [50,51,52].

One-stage fine-tuning enables significant performance improvements but relies heavily on the availability of domain-specific data. The quality and diversity of the medical datasets used in this stage play an important role in the success of fine-tuning [51].

### 3.3. Multi-Stage Fine-Tuning

This staged adaptation method is an expansion of the one-stage methodology that is characterized by including more stages in the fine-tuning of the model to more focused tasks or working with distinct datasets. This approach strengthens the flexibility of the model to work well when used on different medical image analysis problems and makes the model stronger. The progressive approach is particularly beneficial for handling complex datasets that span multiple domains or require understanding different modalities (e.g., combining 2D and 3D medical images), as illustrated in Figure 4(3).

In this phased fine-tuning, it initially trains the model on large, domain-agnostic data. The training goal at this step allows the model to learn global patterns and ideas to apply them to a wide variety of medical imaging tasks. Further phases improve the capability of the model to perform particular things by tuning the model on smaller and more specialized datasets [53,54].

The process of fine-tuning structures the model to be more specific regarding the complexity of medical image analysis in each stage. As an example, one stage may be training the model with a general medical image dataset, and other stages may further refine the model to perform specific tasks such as image segmentation or diagnostic report generation.

The multi-stage fine-tuning has a number of benefits such as better generalization of multiple tasks and compatibility with different types of data. Nevertheless, it is a demanding method that involves careful treatment of the training phases as well as access to comprehensive sets of data. It is also more resource-intensive to perform fine-tuning than zero-stage and one-stage fine-tuning since it requires more training steps [53].

### 3.4. Benefits of X-Stage Tuning

The X-Stage Tuning Paradigm provides a flexible and scalable approach to adapting LLMs for medical image analysis. It allows researchers to select the most appropriate fine-tuning strategy based on each task’s unique demands and the available data. The main benefits of staged fine-tuning include:

- Efficiency: Zero-stage fine-tuning offers a quick deployment of pre-trained models without the need for task-specific data. It is particularly useful when resources are limited or when rapid application of models is required.
- Task-Specific Adaptation: One-stage fine-tuning increases the performance on target medical tasks by fine-tuning the model to the specificities of the medical field.
- Robustness and Flexibility: By using a multi-stage fine-tuning, the model can work with multimodal, complex data and generalize them on a range of tasks. It gives an advantage of flexibility in the application of the model to the various fields of medicine; thus, it is applicable in various and multifaceted clinical surroundings.
- Improved Generalization: Multi-stage fine-tuning improves the generalization and flexibility of LLMs to work with data across many medical domains or modes of data by continually adjusting the model to the practice of multi-stage training [54,55].

Table 2 and Table 3 outlines the X-stage tuning paradigm for LLMs in medical imaging, comparing open-source (OS) approaches. Meanwhile, Table 2 evaluates LLM performance on key medical VQA datasets (VQA-RAD, SLAKE, PathVQA), reporting accuracy for open-ended (O), closed (C), and overall (OA) questions, with top results highlighted in blue.

## 4. Experimental Setup

This survey paper does not conduct new experiments but systematically reviews existing works on LLMs for medical images. The experimental setup is derived from the methodologies of the analyzed studies, which typically follow the steps outlined below.

### 4.1. Selection of Papers, Countrywise and Yearly Roadmap

**Figure 5:**
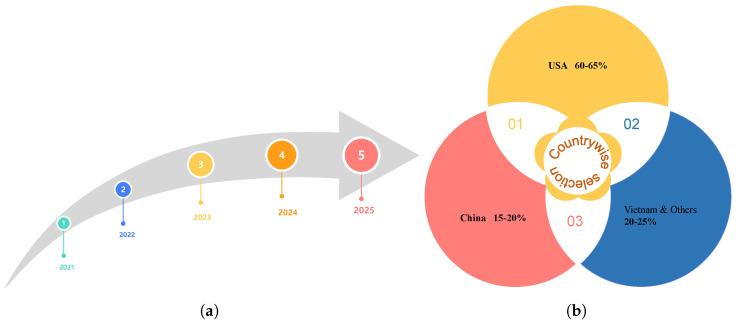
Roadmap of five years (**a**). Countrywise selection (**b**).

This survey covers research papers published between 2021 and 2025, presenting a comprehensive roadmap of recent advancements in the field over the past five years, as illustrated in Figure 5a. The geographic distribution of identified papers reveals that 60–65 % of studies originate from the USA, 15–20 % from China, and 20–25% from other countries, including Vietnam, various European nations, and multinational collaborations, as illustrated in Figure 5b. This breakdown highlights both the temporal progression of research activity and the current concentration of medical LLM research in these two leading AI hubs while underscoring the need for broader global participation in future work.

### 4.2. Model Selection and Tuning Paradigms

We explore model selection and tuning paradigms for large language models (LLMs) in medical imaging, categorizing them into three stages: zero-stage tuning, where pre-trained LLMs (e.g., GPT-4V, Gemini) are applied directly to medical tasks without any fine-tuning; one-stage tuning, where models (e.g., MedBLIP, RadFM) are fine-tuned on domain-specific datasets such as MIMIC-CXR; and multi-stage tuning, where models (e.g., LLaVA-Med, PeFoMed) undergo progressive training on both general and medical datasets. The models were chosen on the capability of supporting complex applications of medical image analysis, especially those with large amounts of data. We used various tuning paradigms to maximize various tasks, like automated diagnosis and report generation, among other tasks, on models. ImageNet, MIMIC-CXR, and MedicalSeg, among other datasets, were used in the training process and were chosen in a way that would facilitate a wide scope of medical imaging difficulties. The given systematic review sheds some light on the performance of every available method, giving a strategic plan of how to move forward in medical AI. By categorizing these paradigms, we aim to offer a deeper understanding of how each tuning method influences model accuracy and adaptability, which is essential for advancing the application of LLMs in healthcare.

### 4.3. Benchmark Tasks and Datasets

**Figure 6:**
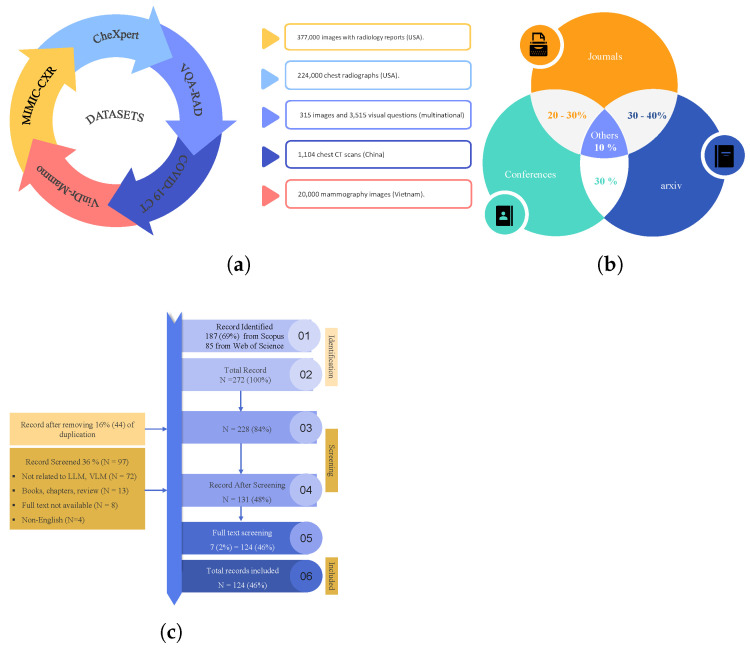
Datasets (**a**), journal research papers and conferences published in the selected years (**b**), PRISMA flow diagram illustrating the systematic literature selection process from Scopus and Web of Science databases (2021–2025). The diagram includes identification, screening, eligibility assessment, and final inclusion of studies, with documented reasons for exclusions at each stage (**c**).

This article includes a systematic survey of LLMs on medical imaging tasks through benchmark research elements. Some of the relevant functions are automated radiology report generation, diagnosis type, visual question answering (VQA), and visual grounding tasks. In Figure 6a, the popular datasets identified in the study include MIMIC-CXR (X-rays with reports), CheXpert (chest radiographs), and VQA-RAD/SLAKE (medical visual QA pairs). With the help of these datasets and the aspect in which they are used in assessing the quality of LLM, this work sheds light on the effectiveness of the models and the way the progress of AI-based medical services may evolve. Additionally, Figure 6b outlines the percentage of identified papers from different journals, coneference, and arxiv. In Figure 6c, it can be seen that we identified 272 papers from Scopus and Web of Science using keywords like “LLM for medical images” and “role of VLM in medical images” after removing duplicates. Abstract screening excluded 141 irrelevant records (books, reviews, chapters, non-English, etc.), followed by full-text screening, which excluded 7 additional papers. The final review included 124 studies, ensuring a rigorous and representative literature synthesis.

### 4.4. Key Tasks in Medical Image Analysis Using LLMs

LLMs have demonstrated significant potential across a variety of medical image analysis tasks. Each of these tasks supports different aspects of medical decision-making, from diagnosis to patient care. Below, we discuss the key tasks to which LLMs are applied:

- Automated Radiology Report Generation: Automated radiology report generation is a critical task where LLMs analyze medical images (such as X-rays, CT scans, or MRIs) and generate detailed text descriptions, identifying abnormalities and making diagnostic suggestions. This task often uses datasets like MIMIC-CXR and CheXpert, where medical reports are paired with imaging data, providing the foundation for training models to produce clinically relevant reports.
- Disease Classification: Disease classification is the process of training models in order to perform classification tasks on medical images, e.g., healthy vs. diseased classification. This activity is crucial in the early diagnosis and detection of the disease, which assists medical personnel in coming up with informed choices. Datasets such as MIMIC-CXR and CheXpert are annotated with images containing diagnostic categories, which can be used to assess the effectiveness of models in practice in clinical settings.
- Visual Question Answering (VQA): The model in VQA tasks is presented with a question and answers concerning an image related to the field of medicine, e.g., what is the diagnosis of an X-ray or an MRI? Such a task involves the combination of visual and text data. Medical image datasets with clinical questions and answers, including VQA-RAD and SLAKE, are useful in training models to produce revealing and accurate responses to clinical questions using visualized information.
- Visual Grounding: Visual grounding tasks require recognition of certain areas in an image given from textual descriptions or queries. This may include an analysis of medical images, such as the detection of tumors or other abnormalities in X-rays or MRIs. The model gives a bounding box or segmentation map to signify the position of the identified region. Datasets like CheXpert and Open-I facilitate such works, providing text annotations and image features that ground particular medical entities.

### 4.5. Datasets Used in Medical Image Analysis with LLMs

The effectiveness of LLMs in medical image analysis is heavily dependent on the datasets used for training, testing, and evaluation. Below are some of the key datasets that have been utilized in the research of LLMs for medical images.

- MIMIC-CXR: The MIMIC-CXR includes more than 370,000 objects (chest X-ray images) with accompanying radiology reports. The prevalent use of this dataset includes disease categorization and report automation, in which tasks include deployment of LLMs to write coherent descriptions of the conditions detected in X-rays. Its size and diversity are good choices of data to train LLMs with in the real-world situation of clinical needs.
- CheXpert: CheXpert is a rather big dataset of chest X-rays with labels of several types of diseases, including, e.g., pneumonia, tuberculosis, and cardiovascular diseases. The data also contain uncertainty labels, and on the basis of the uncertainty labels, the models are trained to be able to deal with the uncertain or unclear situations, so it is a valuable resource related to the disease classification activity. CheXpert is an important database to evaluate the out-of-sample performance of LLMs in numerous clinical circumstances.
- VQA-RAD: VQA-RAD is a special dataset that is used to address medical visual question answering (VQA). It has medical pictures that are accompanied by questions and answers, where the model has to infer the visual contents. VQA-RAD can assist in training models that can process both image and text data to respond to clinical questions, and this can contribute a great deal to training AI-related systems concerning clinical decision support.
- SLAKE: One more important dataset in VQA tasks is SLAKE. It comprises both medical images and question–answer pairs, covering as varied a set of clinical scenarios as possible. The data support answering open-ended and detailed medical questions with LLMs to achieve enhanced medical VQA models.
- Open-I: Open-I is a collection of medical images and question–answer pairs on a wide set of images. It is heavily applied in designing and testing visual question answering systems, especially in those that involve LLMs in reading through both medical images and textual descriptions. It is useful with regard to such models that have to respond to clinical questions with reference to visual information in pictures.

### 4.6. Evaluation Metrics in LLMs for Medical Image Analysis

**Figure 7:**
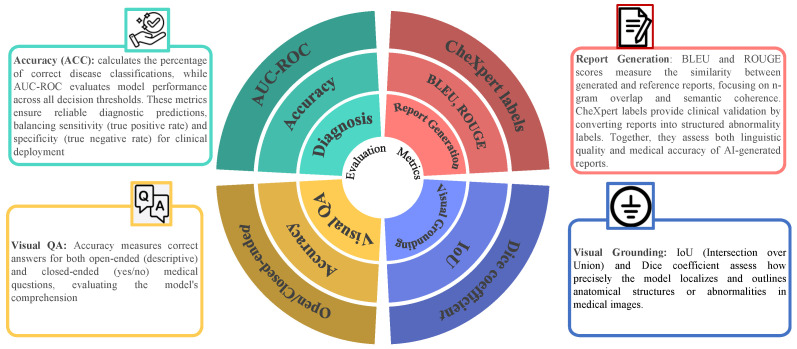
Key datasets used in medical image analysis with LLMs. This includes MIMIC-CXR (chest X-rays with reports), CheXpert (chest radiographs), VQA-RAD/SLAKE (medical visual question answering pairs), and Open-I (a variety of medical images with question-answer pairs). These datasets support various tasks, including disease classification, report generation, visual question answering, and visual grounding.

Evaluation metrics are used for LLMs in medical imaging across key tasks. The evaluative metrics for medical report generation consist of BLEU and ROUGE, together with CheXpert labels for measuring both text quality and clinical relevance. Diagnosis classification is evaluated using accuracy (ACC) and AUC-ROC, ensuring robust predictive performance [25,98,99]. The accuracy metric measures both open- and closed-ended questions in Visual question answering, while visual grounding is measured using IoU and Dice coefficient for spatial alignment. This study provides a comprehensive analysis of these benchmarks to guide future advancements in AI-driven medical applications [100,101,102] as presented in Figure 7. Table 4 sumarizes the evaluation metrics used for different tasks in medical image analysis.

### 4.7. Evaluation of LLMs in Medical Image Analysis

Evaluating LLMs for medical image analysis involves the use of standard metrics to assess performance across different tasks. Common evaluation metrics include:

- Accuracy: Measures the percentage of correct classifications or answers generated by the model. This metric is commonly used in disease classification and VQA tasks.
- F1-Score: A harmonic mean of precision and recall, the F1-score is widely used to evaluate the balance between correctly identified disease cases and false positives/negatives.
- Area Under the Curve (AUC): This metric evaluates the performance of classification models, particularly in tasks with imbalanced datasets. AUC provides a measure of the model’s ability to discriminate between different classes (e.g., disease vs. healthy).
- BLEU and ROUGE: These are text-based evaluation metrics used for automated report generation. BLEU measures the overlap of n-grams between the generated and reference text, while ROUGE focuses on recall-based evaluation of n-grams.
- Intersection over Union (IoU): Used in visual grounding tasks, IoU measures how well the predicted bounding box matches the ground truth. It is a critical metric for evaluating the accuracy of segmentation and localization tasks.

A core contribution of this survey is the synthesis of evaluation metrics used across various LLM-driven medical imaging tasks. Table 4 summarizes which metrics are applied to each task and how they are used.

- Medical Report Generation:
  BLEU: Measures n-gram overlap between generated and reference reports.
  ROUGE: Evaluates recall-based n-gram similarity.
  CheXpert Labels: Converts free-text reports into structured clinical labels for validation.
- Disease Classification:
  Accuracy (ACC): Proportion of correctly predicted diagnostic labels.
  AUC-ROC: Measures the model’s ability to distinguish between classes (e.g., disease vs. healthy).
- Visual Question Answering (VQA):
  Accuracy: Evaluates correctness of responses to open- and closed-ended questions.
  BLEU (for descriptive responses): Measures linguistic quality of generated answers.
- Visual Grounding/Segmentation:
  IoU (Intersection over Union): Measures overlap between predicted and actual regions.
  Dice Coefficient: Measures similarity between predicted and reference segmentations.

These metrics are consistently used across benchmark datasets such as MIMIC-CXR, VQA-RAD, SLAKE, and CheXpert. Their unified synthesis helps standardize future model evaluation across medical imaging tasks.

## 5. Challenges

The adoption and optimization of large language models (LLMs) for medical image analysis necessitate addressing multiple existing challenges. These challenges arise from issues such as data scarcity, the evolving nature of medical knowledge, model output hallucinations, and concerns regarding privacy and security. This section examines these challenges in detail and proposes potential solutions for addressing them. Table 5 presents the key challenges faced when applying LLMs in medical image analysis, describing the impact on model performance and potential solutions for each issue. It highlights the major obstacles that need to be overcome for LLMs to be fully utilized in clinical settings [55,103,104,105].

### 5.1. Lack of Large High-Quality Datasets for Medical Images

The limited availability of extensive and accurate annotated medical image datasets stands as a major barrier in using LLMs for medical image analysis [106]. Training LLMs requires substantial amounts of data, particularly data that are labeled with expert knowledge. However, obtaining medical image datasets with comprehensive annotations is often expensive, time-consuming, and limited by privacy concerns. Many existing medical image datasets are either small in scale or lack the diversity needed to enable robust model development that can generalize across different medical conditions and imaging modalities [107].

To address this challenge, one potential solution is to create synthetic datasets by leveraging techniques like data augmentation or generative models. These methods can be used to generate additional training data, potentially alleviating the data scarcity problem. Another approach is to implement federated learning, which allows models to be trained across multiple institutions without sharing sensitive patient data, thus ensuring both privacy and data diversity [108].

### 5.2. Knowledge Editing in LLMs for Medical Images

The field of medical knowledge evolves all the time, and each day new diseases, disease treatments, and methods of imaging are discovered. Yet, LLMs are usually trained on fixed datasets and cannot experience automatic knowledge update after training [109]. This shortcoming implies that LLMs might not be able to use the most recent medical breakthroughs, thus producing out-of-date or even erroneous findings.

Knowledge editing could be one of the solutions to counter this problem. With knowledge editing, one can create the capability to change the knowledge database of an LLM without as many retraining procedures. Such a strategy will enable LLM to be flexible and embrace the updated medical information. One of the most essential methods of knowledge editing is a continuous learning mechanism where updating of the knowledge base is carried out by the model with little or no retraining. Moreover, integrating external databases, including those that constantly update the repositories of medical literature, enables the LLMs to keep up with the latest advances in medical science. The mechanisms of keeping LLMs up to date with relevant information, as well as keeping them in line with recent developments, also allow the LLMs to adjust to new knowledge in medicine more effectively and offer appropriate advice in the clinic [110].

### 5.3. Hallucination in LLMs for Medical Images

The other important concern in using LLMs in medical image analysis is the problem of hallucinations, in which the models produce inaccurate or misleading results. Such an issue is especially alarming in the medical sphere, where a misdiagnosis or an improper suggestion might be disastrous as far as patient care is concerned. The hallucination arises frequently when the model is uncertain about its predictions or where the input data are unclear, and the interpretation becomes faulty [111].

Hallucinations can be mitigated by a number of strategies. One of them is human feedback (RLHF) tasks in which the model is retrained using expert know-how to fix incorrect responses. This process enhances the precision and consistency of the model by enhancing the comprehension of the model, grounded in practical knowledge. Additionally, integrating external knowledge bases or reasoning modules can help the model generate more reliable and accurate responses by providing contextual support and reinforcing medical knowledge. Regular model evaluation and validation using medical experts play a critical role in detecting and preventing hallucinations, as expert oversight ensures that model outputs align with clinical expectations and are clinically accurate [112,113].

### 5.4. Privacy and Security of Medical Information in LLMs

The use of medical images often involves sensitive patient data, and privacy is a significant concern when training LLMs in healthcare settings. Traditional methods of training models require centralized access to large datasets, which raises the risk of exposing sensitive information. This issue is particularly important when handling medical data, as improper handling or breaches could lead to violations of patient confidentiality [114].

To ensure privacy, methods such as federated learning [115] can be employed. Federated learning allows models to be trained locally at different institutions while keeping the data secure and private. The model aggregates insights from local training without transferring raw data, minimizing the risk of data breaches. Additionally, techniques such as differential privacy can be incorporated to ensure that sensitive information is not inadvertently exposed during the training process.

### 5.5. Model Interpretability and Trustworthiness

Trustworthiness and interpretability of a model are also vital in the medical sphere. Healthcare professionals must be able to interpret the decision tree of LLMs in executing crucial roles in diagnostic and therapeutic planning [116,117]. Some LLMs, particularly large models, however, are used as a black box, which can make it challenging to interpret the decision-making process.

To make LLMs usable in a clinical setting, it is crucial to improve their interpretability. The decision-making of the model can also be made more transparent using methods such as explainable AI (XAI) or saliency mapping which display the image areas that had the greatest effect on the model. Moreover, the models may be modified to offer confidence scores and lines of argument so as to assist healthcare specialists to comprehend why the model makes the predictions.

## 6. Future Directions

As the development of large language models (LLMs) in medical image analysis continues to evolve, a range of interesting opportunities and possible innovations are in the cards. This section explains the major areas of study within which one can focus in the future in order to develop the abilities of the currently available ILM further, as well as solving the limitations present in the current usage. Other streams of research that should be pursued in the future include the combination of vision-language models (VLMs) in medical image interpretation. On the one hand, multimodal data integration between visual, textual, and even audio or temporal data is already an easy route to take, since the VLMs are continuously evolving towards the multimodal scope. Such integration would be able to add a lot of value to clinical decision-making, providing richer, contextually aware information. Also, a more adaptive and dynamic fine-tuning approach still would enable VLMs to adapt to new information and learn continually about new medical knowledge with real-time customized assistance to healthcare practitioners. In addition, the cross-lingual and cross-cultural flexibility will be needed for VLMs so that they can be applicable all over the world without any biases in terms of region and language. Such developments would facilitate the overall contribution of VLMs and LLMs to the change in healthcare.

### 6.1. Unified Evaluation Metrics for Medical Image Tasks

A major issue hindering the advancement of LLMs in medical image analysis is that studies often employ conflicting evaluation metrics. Multiple assessment metrics currently exist for evaluating model performance in automated report creation, disease classification [44], and medical visual question answering. These metrics, including BLEU, ROUGE, accuracy, and F1-score, vary significantly depending on the task and dataset, making comparisons between models challenging and hindering the standardization of progress in the field [118,119,120]. For instance, a model may achieve high accuracy on a disease classification task; however, this could be misleading if the dataset is imbalanced, with a dominant class, such as “healthy” images. In this case, the model might perform poorly on the minority class (e.g., rare diseases) but still achieve a high overall accuracy. Conversely, the F1 score, which takes both precision and recall into account, may reflect a more balanced performance across all classes, showing how the model performs on both the majority and minority classes.

A unified set of evaluation metrics is essential for facilitating fair comparisons and advancing the creation of enhanced models. Subsequent investigations should focus on establishing standardized metrics for various medical image tasks that consider both diagnostic accuracy and text generation quality. This will help streamline model development and enable more consistent and reliable performance evaluation across the field [121,122].

### 6.2. Multilingual Capabilities in LLMs for Medical Images

Currently, most large language models (LLMs) for medical image analysis are primarily trained on English-language datasets. However, healthcare is a global issue, and medical image data from non-English-speaking countries and regions are often underrepresented. The lack of multilingual support in LLMs limits their applicability and usefulness in diverse clinical settings [123]. As medical image analysis continues to expand globally, addressing language barriers is crucial for ensuring that LLMs can be effectively applied across different linguistic and cultural contexts.

To overcome this limitation, it would be worthwhile to consider future studies in establishing multilingual LLMs that can deal with medical image analyses work across several languages. Incorporation of datasets that correspond to multiple languages potentially allows these models to ensure more inclusive healthcare solutions, as well as options that can improve the availability of diagnostic tools in areas with limited access to English content. Multilingual LLMs will help fill the high-resource-low-resource divide in medical diagnostics, allowing medical workers across the globe to access AI-based tools in their respective settings, irrespective of their linguistic or geographical belonging [124].

Also, cross-cultural adaptability concerns the necessity of vision-language models (VLMs) to operate across various linguistic and cultural environments, as well as healthcare system environments. Although the majority of the medical image analysis models are trained on the data of the largely English-speaking world, the healthcare practice, medical terminology, and patient demographics vary significantly around the world. That is why it is necessary to design VLMs that are culture-sensitive and capable of interpreting data within the scope of various medical procedures. VLMs can give fairer and more accurate healthcare support by considering multilingual datasets and accommodating cultural differences in symptoms, diagnosis signs, and treatment strategies. This intercultural resilience can be especially effective in areas where exposure to English-speaking sources are scarce, and health technologies cannot be used to the full extent. By making sure that VLMs can be used regardless of the language and cultural background, one will be able to minimize disparities in the delivery of medical services; thus, the innovative techs will be broader and cross-nationwide.

### 6.3. Integration of LLMs with Medical Decision Support Systems

The best potential of further progress of the research on LLMs can be seen when applied to medical decision support systems (MDSSs). MDSSs can assist medical providers in making use of patient information in terms of clinical recommendations with the assistance of its decision-making capabilities. By means of incorporating LLMs into MDSSs, these systems would be able to gain the ability not only to generate medical reports, but also to provide suggestions for diagnoses and individual treatment plans depending on medical images.

The incorporation of LLMs in MDSSs would render a more efficient workflow in clinics, coupled with offering superior, well-informed decision making to clinicians. Subsequently, work can be performed on creating frameworks where LLMs can be used in a seamless manner, incorporated with existing MDSSs, that would have the capability of providing real-time feedback and decision-making support directly based on medical imaging data [125,126].

### 6.4. Chain-of-Thought (CoT) Reasoning for Medical Image Analysis

LLMs exhibit limited reasoning capacities for complex tasks, especially when analyzing medical images. Chain-of-Thought (CoT) reasoning is a method that can significantly enhance the interpretability and decision-making capabilities of LLMs. CoT reasoning involves breaking down a complex task into a sequence of logical steps, which helps the model produce more reliable and understandable answers. In medical image analysis, CoT reasoning could boost LLMs’ capacity to generate diagnostic explanations, allowing these systems to outline the rationale behind a diagnosis or treatment suggestion. Enhanced trust and reliability in clinical practice become possible through the implementation of CoT reasoning in LLM-based systems [127]. The integration of CoT reasoning into medical image analysis models would enable healthcare professionals to better understand how the model arrives at specific conclusions. By providing a clear and logical breakdown of the decision-making process, CoT reasoning fosters greater transparency, leading to more informed clinical decisions. Future research should focus on methods for integrating CoT reasoning systems to improve both the accuracy and interpretability of LLMs in medical imaging procedures [126,128,129].

#### Dynamic Fine-Tuning for Continuous Learning

One of the strategies is dynamic fine-tuning, where VLMs are constantly updated and fine-tuned using new information. Since medical information and knowledge is fast changing, especially with the entry of new diseases, remedies, and diagnostic procedures, it is imperative that VLMs be kept up to date. Dynamic fine-tuning enables process models to adjust to the latest trends in medicine and patient-specific data so that the system is valuable and feasible with time. This would not be the usual type of training where the models just learn, but in this case, the models would practically become part of the decision-making process through the inclusion of new findings and clinical experiences.

As an illustration, when new imaging modalities/diagnostic markers are discovered, the VLMs can update to be capable of identifying and embracing such findings, making sure that healthcare practitioners are informed about the latest recommendations. Dynamic fine-tuning keeps the medical image analysis models relevant because they constantly learn from the newly available data. This will aid in making the performance of the models sustainable despite the changes occurring in the medical arena, and hence these models will be effective in the clinics when the new learnings are implemented frequently.

### 6.5. Multimodal Learning for Comprehensive Medical Image Analysis

In medical image analysis, the analysis frequently makes use of other kinds of data as well, not just the images, which might include clinical notes, patient history, laboratory results, and genetic information, as well as others. Most existing or available large language models (LLMs) in the medical domain to date have been trained on single-modal data, where either images or just text have been used. This shortcoming limits the extent to which they might reflect a complex, multimodal nature of medical information, which could give a more comprehensive picture of the health of a patient [128].

Improving LLMs by training them to gather and synthesize data across modalities should also be the object of future research. As an example, an additional X-ray image can be much more informative when it is accompanied by clinical notes, lab results, and patient demographics. With this kind of training, LLMs can create insights into patient diagnosis and care options that may be richer and more contextual by drawing on all of these different data points. Such practice can be implemented to enable more specific, individualized healthcare suggestions because models will be able to refer to a larger number of data sources. Multimodal learning will be also able to better simulate clinical human reasoning that can be based on integrating the information of different origins, visual, textual, and contextual when vital decisions need to be made [130,131]. By mimicking this process, LLMs will be able to support healthcare professionals in making more informed, reliable decisions, ultimately improving the quality of care and patient outcomes.

### 6.6. Federated Learning for Privacy-Preserving Medical Image Analysis

Medical image analysis using federated learning is a potentially promising area that could be used to solve privacy issues. Federated learning allows training the models in various institutions without the exchange of sensitive patient data. This will guarantee patient privacy as well as the possibility of different healthcare institutions using the model to enhance their performance [132].

Federated learning may be instrumental in breaking the privacy challenges linked to centralized data aggregation as the use of LLMs in medical image analysis expands. Future research should focus on the best ways to integrate federated learning with LLMs, especially in healthcare, where data security and confidentiality are essential. This would cause a situation whereby the use of LLMs in the clinical setting becomes mainstream and beneficial to all, including the patients [133].

### 6.7. Model Explainability and Trustworthiness

In healthcare, models must have transparency and should be trustworthy to be adopted by clinicians. Large language models (LLMs) in general and deep learning-based ones in particular are usually characterized by being a black box, so it is difficult to explain how they come to specific conclusions. Failure to explain their thought processes makes them less useful in healthcare since reliable AI advice is instrumental in clinical decision-making, where faith in the machine is the most important factor. One way to overcome this difficulty is by enhancing the interpretability of LLMs, where clinicians are able to comprehend and confirm the results of the model.

As potential future work, medical image analysis with LLM should be made explainable by means of saliency mapping, attention mechanisms, and explainable AI (XAI). Saliency mapping shows the parts of an image that are most influential when making the decision in the model, hence the visualization of what features in the image were deemed as essential. By enabling models to concentrate on certain parts of input information, attention mechanisms provide a better understanding of the way in which the model processes the information and prioritizes it as well. XAI methods are used to give a better idea of the processes within the model, so that the decision-making process of a model can be made simpler. Through these methods, clinicians may gain a clearer look at the rationale of the model and what influences its decisions, which will create more trust regarding its outputs. Altogether, an increase in interpretability and transparency of LLMs will not only stimulate their uptake by clinicians but also turn into more trustworthy tools in the practice of healthcare workers. This emphasis on transparency is the key to the production of LLMs that can eventually become an ordinary part of the medical decision-making process, helping to deliver informed, accurate, and positive clinical judgment [134,135].

## 7. Conclusions

In this survey, we mapped the transformational power of large language models (LLMs) as applied to medical image analysis and the associated potential of redefining the clinical workflow and automated diagnostic procedures. The capacity of LLMs to handle both image and text data allows tremendous breakthroughs in image classification, the generation of medical reports, and visual question answering tasks. Multimodal possibilities can be easily combined with the imaging data and textual description, giving better decision support that helps healthcare specialists in the diagnosis and treatment of patients. This incorporation gives a more comprehensive perspective of patient care; therefore, the LLMs are able to improve the quality, efficiency, and personalization of healthcare delivery. The versatility and applicability of LLMs to medical applications are a significant step in increasing the utilization of artificial intelligence in healthcare.

Nevertheless, although the given possibilities seem so exciting, a few issues remain in the utilization of LLMs towards the medical image examination practice. Among the main barriers are the unavailability of good-quality as well as annotated datasets, which are essential in training such models. Most of the available existing datasets that are too small or not diverse enough restrain the possibility of LLMs performing well across different medical conditions and imaging modalities. Also, it is necessary to add new information on medicine to the models continually, otherwise there is a risk of outdated information, and this is a worse problem made by the immutable nature of the current LLMs. Also, problems such as hallucinations, where the model produces reasonable yet incorrect answers, and the interpretability of the model cause concerns about the stability and trustworthiness of LLMs in real clinical settings. Moreover, when working with LLMs in real-world healthcare environments, one of the most critical concerns is the privacy and safety of confidential medical information and a high level of protection and privacy-preserving measures.

Looking ahead, there is considerable potential for overcoming these challenges through ongoing advancements in LLM architectures and training methodologies. Future research must focus on developing more efficient and effective fine-tuning strategies that allow LLMs to adapt better to medical tasks and perform across a range of specialties and domains. Improving the generalizability of LLMs is critical for ensuring their applicability in diverse clinical scenarios. Additionally, enhancing model transparency and interpretability is key to gaining the trust of healthcare providers who need to understand and validate the decisions made by AI systems. A crucial direction for future work is the integration of LLMs with medical decision support systems, which would allow for the real-time application of model outputs to assist clinicians in making informed decisions. Exploring multimodal learning approaches that incorporate other data types, such as electronic health records, genomic data, and clinical notes, will further broaden the scope and utility of LLMs in healthcare.

In conclusion, this survey lays the groundwork for future studies aimed at realizing the full potential of LLMs in medical image analysis. While there are still significant challenges to address, the continued advancements in LLM architectures, the development of more comprehensive datasets, and the integration of LLMs into clinical decision-making systems provide a clear path forward. By addressing these hurdles and leveraging the evolving capabilities of LLMs, we can contribute to more effective, accurate, and personalized patient care. This survey offers the first X-stage tuning framework for LLMs in medical image analysis, helping researchers navigate model adaptation under varying resource constraints. Unlike previous surveys, this work integrates a comprehensive evaluation metric synthesis, comparative tuning strategies, and a vision-language perspective aligned with modern multimodal LLM advances. By addressing current gaps, the survey aims to guide AI developers, support medical system designers, and educate clinicians on leveraging LLMs safely and effectively in real-world workflows. Ultimately, the successful application of LLMs in healthcare promises to revolutionize medical diagnostics, improving outcomes for patients and enhancing the efficiency of healthcare systems worldwide.

## Figures and Tables

**Table 1:** Summary of Large Language Model (LLM) Applications in Medical Image Analysis.

Application	Description	Key Tasks	Challenges
Medical Report Generation	LLMs generate comprehensive medical reports based on medical images (e.g., X-rays, MRIs).	Report generation, text summarization	Data scarcity, accuracy of generated content, hallucinations
Disease Classification	Classifying medical images into diagnostic categories (e.g., identifying disease presence).	Disease detection, diagnostic labeling	Data imbalance, label quality, class overlap
Visual Question Answering	Answering clinical questions based on medical images.	Answer generation, image-text reasoning	Ambiguity in questions, context understanding
Segmentation	LLMs help identify and segment specific regions (e.g., tumors, organs) in medical images.	Image segmentation, anatomical region detection	Precision in boundary detection, multimodal alignment
Multimodal Learning	Integration of image and text data for a unified analysis, improving task performance across domains.	Cross-modal understanding, task integration	Data alignment, computational cost, model complexity
Federated Learning	Privacy-preserving model training across multiple healthcare institutions without sharing data.	Distributed training, model aggregation	Privacy concerns, communication efficiency, model convergence

**Table 2:** Performance of LLM for Medical Images on Representative Downstream Tasks. VQA-RAD, SLAKE, and PathVQA are datasets for medical visual question answering. “O” refers to the accuracy of open-ended questions, “C” represents the accuracy of binary “yes/no” questions, and “OA” indicates the accuracy across all question types. The best results are highlighted in for clarity. **(O = Open, C = Closed, OA = Overall)**.

Type	Model Name	VQA-RAD			SLAKE			PathVQA		
		O	C	OA	O	C	OA	O	C	OA
Zero-stage Tuning	MUMC [56]	71.5	84.2	79.2	79.5	82.4	81.1	39.0	90.4	65.1
	Q2ATransformer [57]	79.2	81.2	80.5	85.1	80.5	82.8	**54.9**	88.9	**74.6**
One-stage Tuning	ELIXR [58]	37.9	69.3	-	73.4	72.1	72.8	68.0	86.2	78.6
	Prefix T.Medical LM [59]	-	-	-	84.3	82.1	83.3	40.0	87.0	63.6
Multi-stage Tuning	MedVInT-TE [60]	69.3	84.2	-	**88.2**	87.7	-	-	-	-
	LLaVA-Med [61]	64.8	83.1	-	87.1	86.8	-	39.6	91.1	-
	MedVInT-TD [60]	73.7	86.8	-	84.5	86.3	85.2	72.2	92.0	79.4
	RadiologyGPT [61]	78.3	86.2	82.2	86.9	85.1	86.0	65.8	93.2	79.1
	PeFoMed [62]	**79.9**	**87.5**	**84.4**	83.1	**88.7**	**85.3**	45.7	**91.3**	68.6

**Table 3:** X-stage Tuning Paradigm for LLMs in Medical Images. Large Vision-Language Model (LVLM), Open Source (OS).

Type	Model Name	Base	No. of Parameters	Base Detail		
				LLM	LVLM	OS
Zero-stage Tuning	ChatCAD [63]	ChatGPT	-	✓	×	✓
	ChatCAD+ [64]	ChatGPT	-	✓	✓	✓
	Flamingo [65]	Flamingo	9/80	-	✓	✓
	Visual Med-Alpaca [66]	LLaMA	7	✓	-	✓
	Med-Flamingo [67]	OpenFlamingo	9	-	✓	✓
	FFA-GPT [68]	LLaMA2	-	✓	-	✓
	MedBLIP [69]	BioMedLM	2.7	✓	-	✓
	RadFM [70]	3D ViT and MedLLaMA	14	✓	-	✓
	RadFM [70]	LLaMA as LLM	-	-	✓	✓
	GPT-4V [5,71,72]	GPT-4V	-	✓	×	✓
	Gemini [73]	Gemini	-	✓	×	✓
	VisionGPT	VisionTransformer	-	✓	×	✓
One-stage Tuning	OphGLM [74]	ChatGLM	6.2	✓	✓	✓
	Visual Med-Alpaca [66]	LLaMA	7	✓	✓	✓
	FFA-GPT [68]	LLaMA2 [75]	-	✓	✓	✓
	MAIRA-1 [76]	LLaVA-1.5	7	-	✓	✓
	PneumoLLM [77]	CLIP and LLaMA-7	7	-	✓	✓
	R2GenGPT [78]	Swin Transformer and LLaMA2	7	-	✓	✓
	Med-PaLM M [79]	PaLM-E	2/84/562	-	✓	✓
	Prefix T.Medical LM [59]	CLIP and BioGPT	-	-	✓	✓
	FM for MRG [80]	BLIP-2	7.3	-	✓	✓
	MedXchat [81]	CLIP and LLaMA	-	-	✓	✓
	MedVInT [60]	PMC-CLIP and PMC-LLaMA	-	-	✓	✓
	MedBLIP [69,82]	EVA, FLAN-T5/BioGPT	3.4/1.5/2.7	✓	✓	✓
	BioMedGPT	BioGPT	5.2	✓	✓	✓
	RadiologyGPT	RadiologyTransformer	4.5	✓	✓	✓
Multi-stage Tuning	PeFoMed [62]	MiniGPT-v2	7	✓	✓	✓
	SkinGPT-4 [83]	MiniGPT-4 [84]	-	✓	✓	✓
	ELIXR [58]	ELIXR-C: CLIP ELIXR-B: BLIP-2	-	-	✓	✓
	LLaVA-Med [61]	LLaVA	7	-	✓	✓
	CXR-BLIP [85]	BLIP-2 (EVA and OPT Transformer)	2.7	-	✓	✓
	Qilin-Med-VL [86]	ViT and Chiese-LLaMA2-13B-Chat	13	-	✓	✓
	LLM-CXR [87]	dolly-v2-3b	3	✓	✓	✓
	CephGPT-4 [88]	MiniGPT-4	7	✓	✓	✓
	Med-MLLM [89]	ResNet-50 and Transformer	8.9	-	✓	✓
	LMM for RRG [90]	ResNet-50 and GPT2-S	7	-	✓	✓
	XrayGPT [26,91]	MedCLIP and Vicuna	-	-	✓	✓
	PathAsst [92]	LLaVA	13	-	✓	✓
	MOSS-MED [93]	LLaMA2-7B-chat as LLM	7	-	✓	✓
	ClinicalBLIP [94]	InstructBLIP	-	-	✓	✓
	LLIM for MIC [95]	BLIP-2	-	-	✓	✓
	RadMed-Tuning	Med-Tuning	6.0	✓	✓	✓
	LiteGPT [96]	MiniGPT-v2	7	-	✓	✓
	MiniGPT-Med [97]	MiniGPT-v2	7	-	✓	✓
	MedGPT-3	GPT-3	6.5	✓	✓	✓

**Table 4:** The evaluation metrics used for different tasks in medical image analysis.

Task	Evaluation Metric	Description	References
Medical Report Generation	BLEU (Bilingual Evaluation Understudy)	Measures n-gram overlap between generated and reference text to assess fluency and informativeness	[25,98]
- - -	ROUGE (Recall-Oriented Understudy for Gisting)	Measures recall-based n-gram overlap between generated text and reference text	[25,98]
- - -	CheXpert Labels	Assesses clinical relevance of generated text based on medical domain-specific labels	[99]
Disease Classification	Accuracy (ACC)	Measures the overall percentage of correct predictions (e.g., disease vs. healthy)	[100]
- - -	AUC-ROC (Area Under Receiver Operating Curve)	Measures the ability of the model to distinguish between classes (e.g., diseased vs. healthy)	[100]
Visual Question Answering	Accuracy (ACC)	Measures the model’s ability to answer open- and closed-ended questions related to medical images	[101]
Visual Grounding	Intersection over Union (IoU)	Measures overlap between predicted and ground truth regions in image segmentation	[102]
- - -	Dice Coefficient	Measures the similarity between predicted and ground truth regions for segmentation tasks	[102]

**Table 5:** Key Challenges in Using Large Language Models for Medical Image Analysis.

Challenge	Description	Impact on Model Performance	Potential Solutions	limitations
Data Scarcity	Limited access to large, labeled datasets for medical images.	Hinders training and fine-tuning for medical tasks.	Data augmentation, federated learning, transfer learning.	Difficulty in obtaining annotated datasets for rare diseases.
Hallucinations in Generated Content	LLMs may generate plausible-sounding but incorrect medical information.	Decreases model reliability in clinical applications.	Reinforcement learning with human feedback (RLHF), continuous updates.	High risk of generating incorrect diagnoses due to unclear data.
Model Interpretability	Difficulty in explaining model predictions, especially for complex medical scenarios.	Reduces trust in AI-based systems, particularly in clinical use.	Explainable AI (XAI) methods, saliency mapping.	Lack of transparency makes clinical decision-making difficult.
Privacy and Security	Protecting patient data during training, especially when using sensitive medical data.	Legal and ethical issues, data-sharing restrictions.	Differential privacy, federated learning.	Privacy concerns remain for non-centralized data.
Multilingual Model Development	Lack of support for non-English medical image datasets and language diversity in clinical settings.	Limits applicability in non-English speaking regions.	Development of multilingual models, cross-lingual data augmentation.	Limited availability of diverse multilingual datasets.

## Data Availability

Data is contained within the article.
